# Impact of Fiber Structure on the Material Stability and Rupture Mechanisms of Coronary Atherosclerotic Plaques

**DOI:** 10.1007/s10439-017-1827-3

**Published:** 2017-03-30

**Authors:** Graeham R. Douglas, Adam J. Brown, Jonathan H. Gillard, Martin R. Bennett, Michael P. F. Sutcliffe, Zhongzhao Teng

**Affiliations:** 1 Department of Engineering, University of Cambridge, Trumpington Street, Cambridge, CB2 1PZ UK; 2 Division of Cardiovascular Medicine, University of Cambridge, Cambridge, UK; 3 Department of Radiology, School of Clinical Medicine, University of Cambridge, Box 218, Cambridge Biomedical Campus, Cambridge, CB2 0QQ UK

**Keywords:** Coronary, Atherosclerosis, Rupture, Fiber, Stress

## Abstract

The rupture of an atherosclerotic plaque in the coronary circulation remains the main cause of heart attack. As a fiber-oriented structure, the fiber structure, in particular in the fibrous cap (FC), may affect both loading and material strength in the plaque. However, the role of fiber orientation and dispersion in plaque rupture is unclear. Local orientation and dispersion of fibers were calculated for the shoulder regions, mid FC, and regions with intimal thickening (IT) from histological images of 16 human coronary atherosclerotic lesions. Finite element analysis was performed to assess the effect of these properties on mechanical conditions. Fibers in shoulder regions had markedly reduced alignment (Median [interquartile range] 12.9° [6.6, 18.0], *p* < 0.05) compared with those in mid FC (6.1° [5.5, 9.0]) and IT regions (6.7° [5.1, 8.6]). Fiber dispersion was highest in shoulders (0.150 [0.121, 0.192]), intermediate in IT (0.119 [0.103, 0.144]), and lowest in mid FC regions (0.093 [0.081, 0.105], *p* < 0.05). When anisotropic properties were considered, stresses were significantly higher for the mid FC (*p* = 0.030) and IT regions (*p* = 0.002) and no difference was found for the shoulder or global regions. Shear (sliding) stress between fibers in each region and their proportion of maximum principal stress were: shoulder (25.8 kPa [17.1, 41.2], 12.4%), mid FC (13.9 kPa [5.8, 29.6], 13.8%), and IT (36.5 kPa [25.9, 47.3], 15.5%). Fiber structure within the FC has a marked effect on principal stresses, resulting in considerable shear stress between fibers. Fiber structure including orientation and dispersion may determine mechanical strength and thus rupture of atherosclerotic plaques.

**Electronic supplementary material:**

The online version of this article (doi:10.1007/s10439-017-1827-3) contains supplementary material, which is available to authorized users.

## Introduction

Heart disease remains the first global cause of death and the number is expected to rise to more than 23.6 million each year by 2030.^26^ Deaths from heart disease are mainly due to coronary atherosclerotic disease.^49^ Autopsy studies demonstrated that many myocardial infarctions were caused by rupture of a coronary atherosclerotic plaque exhibiting a large lipid-rich necrotic core and luminal thrombus.^48^ The proposed precursor lesion for rupture displays a similar architecture of a thin fibrous cap (FC) separating the lipid core from the lumen, termed a thin-cap fibroatheroma (TCFA).^6^ Clinical and angiographic imaging, including computerized tomography and digital subtract angiography, cannot identify TCFA or other higher risk plaques,^3^ indicating a major need for diagnostic modalities that can identify and stratify higher-risk plaques prior to rupture.

Prospective studies utilizing virtual histology intravascular ultrasound (VH-IVUS)-defined plaque classification algorithms, including PROSPECT (A prospective natural-history study of coronary atherosclerosis), Virtual Histology in Vulnerable Atherosclerosis (VIVA), and ATHEROREMO-IVUS, have shown an association between TCFA and subsequent major adverse cardiovascular events.^7^,^10^,^37^ Although these studies suggest that VH-IVUS can prospectively identify higher-risk plaques, the absolute event rate per lesion identified as higher-risk was low (<10% at 3 years). Thus, additional methods for assessment of plaque vulnerability are required to improve our ability to predict plaque rupture including higher risk features captured by other imaging modalities, e.g., optical coherence tomography.^36^,^50^

Under physiological conditions, atherosclerotic lesion is subject to mechanical loading induced by blood pressure^38^,^42^ and flow. Experimental and computational studies have shed light to the importance of endothelial shear stress in progressive atherosclerosis and rupture. Some of these studies have been summarized elsewhere.^46^ On the other hand, high structural stress concentration within the plaque, which is about 10^3^–10^5^ times higher than endothelial shear stress, has been shown to be associated with fissuring and rupture in both coronary^29^ and carotid^38^ plaques. Structural stress and stretch may also differentiate patient clinical presentations,^15^,^41^,^53^ and are associated with subsequent cerebrovascular^32^,^44^ and heart ischemic events in symptomatic patients.^5^ In general, stable lesions are protected by a thick FC, as increasing FC thickness results in decreasing structural stress.^25^,^52^ Autopsy studies have shown that around 60% of FC ruptures are located in the plaque shoulder region, where luminal curvature is sharper.^29^ Failure at the shoulder can be partially explained by pronounced luminal angulation leading to an increased stress level in the FC.^43^ However, as an anisotropic material with collagen and elastin fibers the primary mechanical components,^8^,^20^,^34^ the fine structures within the FC may be an important determinant in FC stability.^39^ Advancing states of atherosclerosis are known to alter the structure, organization, and concentration of collagen fibers within the tissue matrix.^34^ However, the impact from changes in fiber structure on critical mechanical conditions within atherosclerosis remains unexplored.

Within the cross-section of a healthy artery, fibers are concentric and have a primary orientation aligned with the local lumen tangent.^17^ Atherosclerotic progression often results in an eccentric, non-circular lumen along with disruption to fiber patterns from lipid deposits and intraplaque hemorrhage. In this study, orientation of predominately collagen fibers was extracted from histological images of coronary atherosclerotic plaques. Fiber misalignment was calculated as the local orientation relative to the luminal tangent. The local distribution of fiber orientation relative to their primary orientation was used to calculate fiber dispersion. Fiber misalignment and dispersion were compared between anatomical plaque regions. Two-dimensional finite element (FE) models were reconstructed using the geometry and fiber orientation from the histological images, with loading from an assumed static internal blood pressure. To suggest novel mechanisms for FC failure, mechanical stress was decomposed into components along (axial), perpendicular to (transverse), and shearing between the fibers. The relationship between critical mechanical conditions and fiber architecture was explored.

## Materials and Methods

### Specimen and Histological Processing

Arteries with atherosclerotic plaques were harvested from human hearts during autopsy in consultation with a senior pathologist. The study protocol was approved by the Cambridgeshire Research and Ethics Committee (Ref. 07/H0306/123) and consent obtained from relatives. Sixteen left anterior descending arteries were dissected, excised, sectioned, and stained with hematoxylin and eosin (H&E) and Verhoeff’s Van Gieson (EVG). More details about the specimens and histological processing can be found in our previous report.^4^ Histology slides were digitized using Nanozoomer (Hamamatsu Photonics KK, Hamamatsu, Japan) under 40× magnification.

### Image Processing

**Figure 1:**
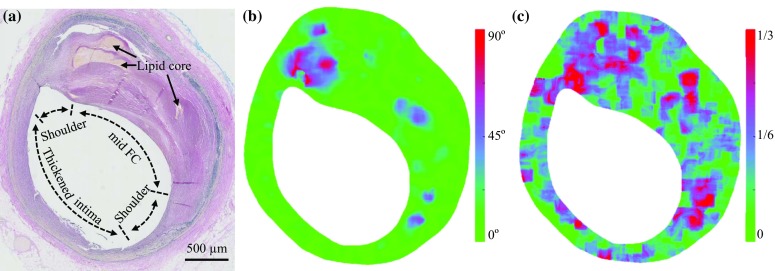
Representative histology image and corresponding distribution of fiber orientation and dispersion ((a) EVG-stained image labelled by tissue regions [Shoulder, mid FC, and region with intima thickening (IT)]; (b) Map of fiber orientation [greener color: better alignment with lumen]; and (c) Map of fiber dispersion [greener color: less fiber dispersion]).

Histological slides from the proximal section of each plaque, where most ruptures occur, were carefully reviewed, and the best slide, without serious artefacts and damage induced by histological processing, was included in the analysis. Image segmentation was performed by an experienced cardiac pathologist to identify atherosclerotic plaque components, including FC and lipid (Fig. 1a). The region adjacent to the lumen was divided into sub-regions of shoulder, mid FC, and intima thickening (IT) to perform location-dependent analyses (Fig. 1a). In this study, unless otherwise specified, FC included fibrous tissues in the shoulder region and mid FC.

**Figure 2:**
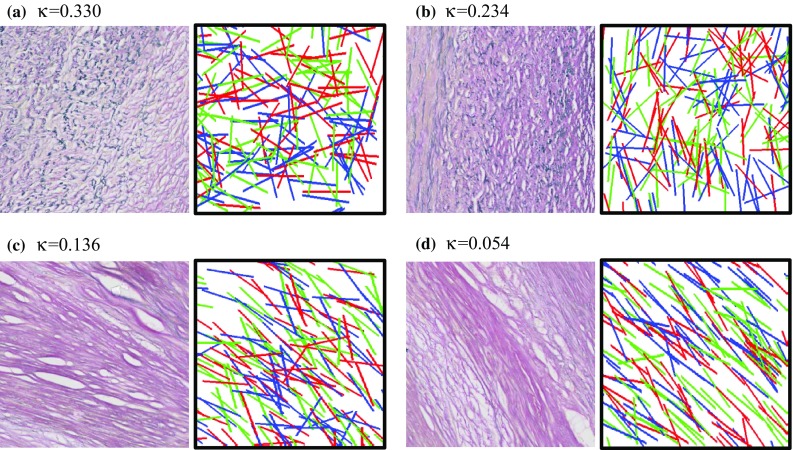
Histology enlargement and generated plot of synthetic fibers with the same orientation and dispersion ((a) a region with nearly even distribution of fiber orientation; (b) a region with less evenly distributed fiber orientation; (c) a region with relatively less spread in fiber orientation; and (d) a region with very little spread in fiber orientation; $\kappa \in [0, 1/3]$ indicates the extent of fiber dispersion, larger $\kappa$ for more misalignment of fibers).

Each image was exported from NDP.view 2.3.0 (Hamamatsu Photonics KK, Hamamatsu, Japan) in bitmap format with pixel size of 3.5 × 3.5 *µ*m^2^ in preparation for further processing to quantify the fiber orientation and dispersion (Figs. 1 and 2). Fibers were assumed to be slender structures with strong contrast against their background and were identified as edges in the image. Details of methods used to identify and to calculate the orientation and dispersion (i.e. the spread) of fibers are included in the Supplemental Material.

### Finite Element Analysis

Segmented contours of the lumen, lipid region, and outer edge of the plaque were used to construct two-dimensional (2D) FE models for each image. Two comparative FE models were built. First, an isotropic analysis assumed that fibers were evenly scattered in all directions. Second an anisotropic analysis assigned fiber orientation by element.

The fibrous tissue was modeled as a Gasser-Ogden-Holzapfel anisotropic, incompressible hyperelastic solid,^17^ defined by the strain energy density function, *ψ*:

$$ \psi = C_{1} \left( {I_{1} - 3} \right) + \frac{{k_{1} }}{{2k_{2} }}\left\{ {\exp \left[ {k_{2} \left( {\kappa I_{1} + \left( {1 - 3\kappa } \right)I_{4} - 1} \right)^{2} } \right] - 1} \right\} $$

in which $I_{1}$ is the first invariant of the deformation tensor. The fiber structure is described by invariant $I_{4} = \left( {\lambda_{1} \cos \alpha } \right)^{2} + \left( {\lambda_{2} \sin \alpha } \right)^{2}$, where $\alpha$ is the fiber orientation within the cross-sectional plane of the FE model, and λ_1_ and λ_2_ are the stretch ratios along and perpendicular to the local fiber orientation, respectively. Since fiber orientation was assigned by element, fiber dispersion was accounted for by the local value of $\alpha$ as well as a material fiber dispersion of $\kappa = 0.136$. The remaining parameters are material constants that used values from a previous study for carotid plaque tissue^19^: the linear isotropic stiffness of the ground matrix is $C_{1} = 0.056$ MPa; the strain-stiffening response of the fibers is described by $k_{1} = 41.08$ MPa and $k_{2} = 1749.6$.

The lipid tissue was modeled as an isotropic, incompressible hyperelastic solid with a reduced polynomial strain energy density function, $\phi$:

$$ \phi = A_{1} \left( {I_{1} - 3} \right) + A_{2} \left( {I_{1} - 3} \right)^{2} $$

in which $A_{1} = 0.0225$ MPa and $A_{2} = 0.2150$ MPa, obtained through fitting uniaxial-extension stress-stretch curves from lipid tissues strips from human carotid atheromas.^45^

**Figure 3:**
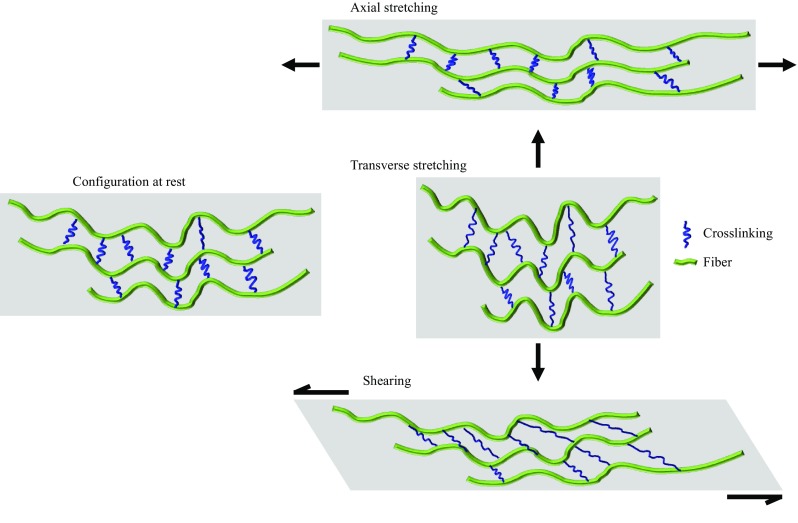
Schematic drawing of a fibrous material under different loading ((a) at rest; (b) stress along the fibers (axial stress); (c) stress perpendicular to the fibers (transverse stress); and (d) stress shearing the fibers (shear stress)).

The geometry was meshed using edge seeds of 5.0 *µ*m on the lumen, 10.0 *µ*m on the lipid edge, and 50.0 *µ*m on the outer edge using the Abaqus FE package (Abaqus 6.13-2, Dassault Systèmes, France). The leftmost point of the outer wall was fixed to prevent rigid body movements and an internal pressure of 16 kPa (120 mmHg) was applied to the lumen surface. Unrealistically high stress might be calculated in FE models from sharp geometries or fiber discontinuities. Therefore, reported stress for each segmented region was the stress that at least 2500 μm^2^ of the region surpassed. This filter ignored about 15–30 highest-stress elements in each region. Isotropic maximum principle stress (Stress-P_1_) from the isotropic model, and anisotropic Stress-P_1_, axial, transverse, and shear stress from the anisotropic model were extracted for analysis. A schematic drawing for each stress component is shown in Fig. 3.

### Statistical Analysis

A paired *t* test or Wilcoxon-Mann–Whitney test was used where appropriate to assess the difference in fiber orientation, dispersion, and critical mechanical conditions between different plaque regions. A significant difference was assumed if *p* < 0.05.

Simple linear regressions explored the relationship between fiber characteristics (fiber misalignment and dispersion), local geometrical factors, and finite element results, including isotropic Stress-P_1_, anisotropic Stress-P_1_, axial, transverse, and shear stress, for regions around the lumen. These models report the unit-dependent slope of the regression; the p-value for this slope being zero (or no statistical relationship); and a regression coefficient (R^2^) that describes how much of the output variability is predicted by the independent parameter. As there are complex interdependencies between geometry, fiber structure, and mechanics, the regression coefficient can be small for significant relationships (*p* < 0.05) between variables. Geometry parameters considered in this study are defined in Table 1.

**Table 1:** Definition of geometry properties used in linear modelling.

Parameter name	Description
Plaque burden (%)	Proportion of plaque area to total area within the external elastic lamella (EEL: boundary between media and adventitia)
Plaque area (mm^2^)	Area of tissue within the EEL excluding the lumen
Lumen area (mm^2^)	Area within the artery wall
Lipid area (mm^2^)	Area of the manually segmented lipid-rich core
Lipid fraction (%)	Proportion of lipid area to total area with the EEL
Lipid thickness (mm)	Maximum radial thickness of the lipid-rich core
Lipid arc (°)	Maximum circumferential arc of the lipid-rich core
FC thickness (mm)	Minimum radial thickness of the fibrous cap
Shoulder curvature (mm^−1^)	Inverse of the median radius of lumen curvature at the shoulder regions. A higher curvature indicates sharper geometry. (For linear regressions within the shoulder region, the same shoulder was used for curvature as for the fiber or FE result being modelled. For regressions within the mid FC and IT, the lower of the two shoulders’ curvature was used)
Normalized shoulder curvature	The shoulder curvature, normalized by the diameter of the EEL in the vertical direction of the plaque image

Multivariate regressions analyzed these complex interdependencies to better predict anisotropic stresses. From an initial regression of isotropic Stress-P_1_ against an anisotropic stresses, geometry and fiber parameters were added to the regression if doing so increased the R^2^ by at least 0.1. All statistical and regression analyses were performed in Matlab R2016a (The MathWorks, Inc., USA).

## Results

### Fiber Orientation and Dispersion

As shown in the illustrative plaque (Fig. 1a), fibers in the narrow region adjacent to the lumen boundary were predominately oriented parallel to the boundary (Fig. 1b), although the orientation deeper in the plaque is less aligned with the lumen. In contrast, fibers in the shoulder region were not well aligned and in some cases were perpendicular to the local lumen edge (Fig. 1b). Quantification of orientation showed better alignment of fibers with the lumen boundary in the mid FC and IT than the shoulder region (Table 2). Dispersion was highest in the shoulders, intermediate in the IT region, and lowest in the mid FC (Fig. 1c and Table 2). For reference, a fiber dispersion of $\kappa = 1/3$ is an evenly scattered set of fiber orientations (the local material is isotropic) and $\kappa = 0$ indicates that all fibers are parallel. Figure 2 shows a series of magnified histological sections across a range of fiber dispersions, paired with plots of synthetic fiber having the same fiber orientation and dispersion.

**Table 2:** Fiber orientation and dispersion in the regions along the lumen (data presented as median [interquartile range]).

	Shoulder	Mid FC	IT
Fiber orientation	12.9° [6.6, 18.0]*	6.1° [5.5, 9.0]	6.7° [5.1, 8.6]
Fiber dispersion	0.150 [0.121, 0.192]	0.093 [0.081, 0.105]^†^	0.119 [0.103, 0.144] ^‡^

*Fibers in the shoulder region had more misalignment than those either in the mid FC or IT (*p* < 0.05)

^†^Fibers in the mid FC had less dispersion than those in either the shoulders or IT (*p* < 0.05)

^‡^Fibers in the IT region had less dispersion than those in the shoulders (*p* < 0.05)

### Differences in Stress-P_1_ Between Isotropic and Anisotropic Models

**Figure 4:**
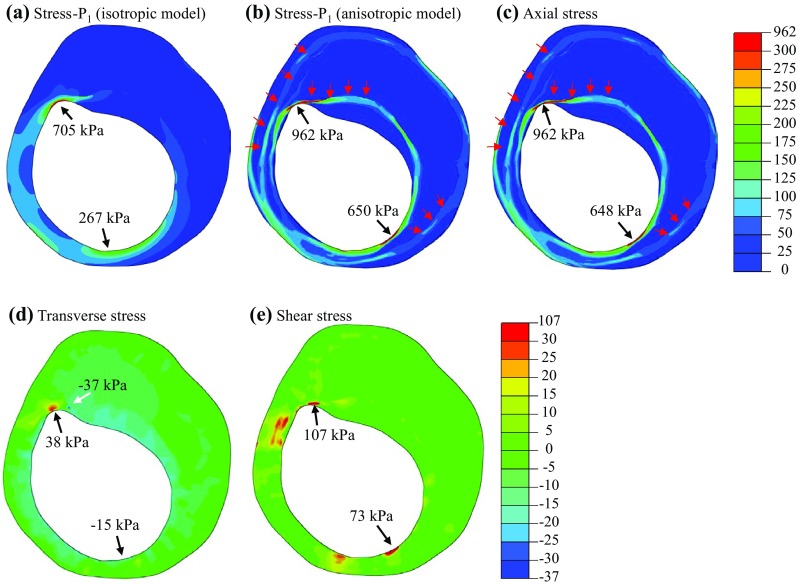
Band plot of stress under lumen pressure ((a) Stress-P_1_ predicted using the isotropic model; (b) Stress-P_1_ predicted using the anisotropic model; (c) Stress along the fibers [axial stress]; (d) Stress perpendicular to the fibers [transverse stress]; and (e) Stress shearing the fibers [shear stress]; red arrows in B and C identify slender bands of high stress).

**Figure 5:**
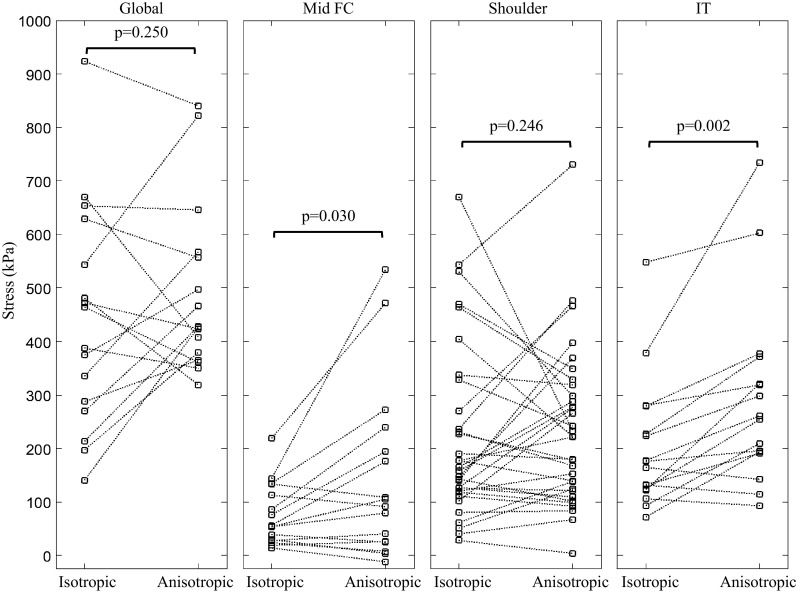
Comparison of Stress-P_1_ between the isotropic and anisotropic models, compared globally and in different plaque regions (Global peak stress and the stress value in the shoulder region calculated by both models were comparable; the stress values in mid FC and IT were underestimated by the isotropic model).

Representative maximum principal stress (Stress-P_1_) plots from isotropic and anisotropic models are shown in Fig. 4a and 4b. Stresses were usually higher near the lumen in both models. In the isotropic models, the stresses were often highest and concentrated in the shoulder regions, where luminal curvature was tighter. In the anisotropic models, higher stress zones formed slender bands, which extended deeper into the tissue towards and diverged around the lipid core (Fig. 4b; Supplemental Fig. S1). We examined global and regional stress using both isotropic and anisotropic models. There was no significant difference between the isotropic and anisotropic models for global Stress-P_1_ (Fig. 5 and Table 3). In sub-regions along the lumen, significant differences in Stress-P_1_ between the two models were found for the IT regions and in the mid FC, but not for the shoulder regions. Stress-P_1_ was higher for the shoulder compared to the mid FC regions in 15/16 of the isotropic models and 14/16 of the anisotropic models.

**Table 3:** Region-by-region comparison of maximum principal stress (Stress-P_1_) between the isotropic and anisotropic models (median, [interquartile range]; unit: kPa).

	Isotropic Stress-P_1_	Anisotropic Stress-P_1_	*p* value
Global peak stress	426.3 [278.8, 586.4]	427.8 [372.5, 562.0]	0.250
Mid FC	55.1 [28.6, 123.2]	98.4 [25.6, 217.2]	0.030
Shoulder	161.4 [119.5, 299.2]	221.9 [122.9, 308.6]	0.246
IT	170.4 [123.8, 253.2]	257.9 [192.6, 346.8]	0.002

### Axial, Transverse, and Shear Stress Derived from the Anisotropic Model

**Figure 6:**
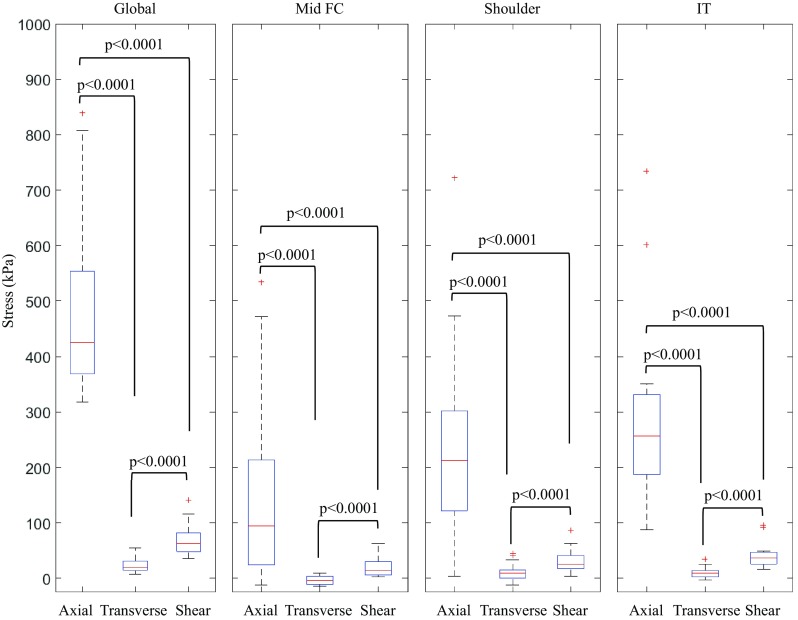
Box-plot of different stress components (axial, transverse and shear stresses) globally and in different plaque regions (The red line marked the median value; upper and bottom edges of the box showed the 1st and 3rd quartile; the whiskers were for 1.5 times the interquartile range, and points outside the whiskers were marked individually as crosses.).

Anisotropic models had components of stress along fibers (axial stress), across fibers (transverse stress), and sliding between fibers (shear stress) (Fig. 4 and Table 4). For clarity, the deformation of fibers and their crosslinks produced by these stresses is illustrated by a cartoon (Fig. 3). Plaques mainly undertake mechanical loading along consecutive fibers, as indicated by axial stress being the largest component (Fig. 4 and Table 4). Higher axial stress was found in the shoulder and IT regions than in the mid FC. Both transverse and shear stresses in each sub-region was significantly lower than the axial stresses (Fig. 6 and Table 4). Transverse stresses in the mid FC, shoulder, and IT regions were all significantly lower than their respective shear stresses. Transverse stress was mostly negative (compressive) around the lumen. Considerable shear stress existed in the regions around the lumen as a proportion of the respective region’s peak Stress-P_1_: mid FC (13.8% [0.10%, 27.4%]), shoulder (12.4% [8.8%, 23.2%]), and IT (15.5% [10.1%, 19.6%]). Anisotropic stress components were higher in the shoulder regions than the mid FC for most samples: axial–14/16, transverse–16/16 including one tied, and shear–14/16.

**Table 4:** Region-by-region comparison of components of anisotropic stress (median, [interquartile range]; unit: kPa).

	Axial stress^†^	Transverse stress	Shear stress^‡^
Mid FC	94.2 [24.6, 213.4]	−3.9 [−11.3, 4.0]	13.9 [5.8, 29.6]
Shoulder	212.2 [122.3, 302.3]*	9.6 [0.3, 15.2]	25.8 [17.1, 41.2]
IT	256.9 [187.9, 332.0]*	9.8 [2.9, 13.7]	36.5 [25.9, 47.3]

^†^Axial stresses for a respective region were significantly higher than transverse or shear stresses for the same region (*p* < 0.0001)

^‡^Shear stresses for a respective region were significantly higher than transverse stresses for the same region (*p* < 0.0001)

*Axial stresses in the shoulder and IT were significantly higher than in the mid FC (*p* < 0.05)

### Geometry Parameters and Fiber Structure

Only regressions finding significant associations between geometry parameters and fiber structures are reported in Table 5. Fiber misalignment in the shoulder regions was significantly linked to increases in the normalized shoulder curvature, plaque burden, and FC thickness. Fiber dispersion in the shoulders was predicted by increased normalized shoulder curvature and decreased FC thickness. Higher FC thickness was the only predictor of fiber misalignment in the mid FC, and lower lipid fraction the only link with dispersion. No significant links between fiber structure and geometry parameters were found for the IT region.

**Table 5:** Regression models of geometry parameters against fiber structure by region.

Fiber parameter	Geometry parameter	Mid FC			Shoulder		
		Slope	R^2^	*p* value	Slope	R^2^	*p* value
Fiber orientation (°)	FC thickness	18.5	0.423	0.006	31.0	0.185	0.014
	Plaque burden	–	–	–	30.0	0.161	0.023
	Normalized shoulder curvature	–	–	–	0.471	0.163	0.022
Fiber dispersion	FC thickness	–	–	–	−0.164	0.168	0.020
	Normalized shoulder curvature	–	–	–	0.003	0.165	0.021
	Lipid fraction	−0.124	0.325	0.021	–	–	–

### Geometry Parameters and Finite Element Analysis Results

For the shoulder region, regressions linked increasing isotropic Stress-P_1_ to increasing shoulder curvature and normalized shoulder curvature (Table 6). Transverse stresses increased with shoulder curvature and normalized shoulder curvature. No significant links were found between geometry and anisotropic Stress-P_1_, axial stress, or shear stresses in the shoulder region. For the mid FC, regressions found stresses of all reported types decreased with shoulder curvature and normalized shoulder curvature. For all stress types except shear, stresses increased for wider lipid arcs. Lipid arc is similar to the length of the FC. Shear stress increased with lipid area and lumen area. No significant links between geometry parameters and FE results were found for the IT region.

**Table 6:** Regression models of geometry parameters against finite element analysis results by region.

FE result	Geometry parameter	Mid FC			Shoulder		
		Slope	R^2^	*p* value	Slope	R^2^	*p* value
Isotropic Stress-P_1_	Normalized shoulder curvature	−12.1	0.420	0.007	14.7	0.328	0.001
	Shoulder curvature	−38.6	0.408	0.008	54.7	0.366	<0.001
	Lipid arc	0.597	0.472	0.003	–	–	–
Anisotropic Stress-P_1_	Normalized shoulder curvature	−30.0	0.324	0.021	–	–	–
	Shoulder curvature	−100.8	0.350	0.016	–	–	–
	Lipid arc	1.38	0.316	0.024			
Axial Stress	Normalized shoulder curvature	−29.7	0.318	0.023	–	–	–
	Shoulder curvature	−99.8	0.343	0.017	–	–	–
	Lipid arc	1.38	0.315	0.024	–	–	–
Transverse Stress	Normalized shoulder curvature	−1.76	0.390	0.010	1.43	0.449	<0.001
	Shoulder curvature	−5.93	0.423	0.006	5.10	0.459	<0.001
	Lipid arc	0.085	0.418	0.007	–	–	–
Shear Stress	Normalized shoulder curvature	−3.06	0.303	0.027	1.07	0.133	0.040
	Shoulder curvature	−11.3	0.396	0.009	–	–	–
	Lumen area	7.29	0.291	0.031	–	–	–
	Lipid area	88.0	0.279	0.035	–	–	–

### Fiber Structure and Finite Element Analysis Results

Simple linear regressions only found one statistically significant coupling between fiber structures (misalignment and dispersion) and FE results. Increased dispersion was a predictor of transverse stress in the shoulders (slope = 153.3, R^2^ = 0.216, *p* = 0.007). No other fiber structure parameter was significantly linked to FE results.

### Isotropic Principle Stress and Other Stresses

As the isotropic FE models are a function of geometry and material properties only (i.e. does not have fiber structure), isotropic Stress-P_1_ was also considered as a variable for linear regressions against the anisotropic FE. Thus, results of the regression imply the degree to which fiber structure affects anisotropic stresses. Regressions indicated that isotropic Stress-P_1_ was a significant predictor for anisotropic stresses in all regions, except shear and transverse stress in the IT region (Table 7). Isotropic Stress-P_1_ alone explains the majority of anisotropic Stress-P_1_ and axial stress variability in the mid FC and IT regions (R^2^ = 0.695–0.712).

**Table 7:** Regression models of isotropic Stress-P_1_ against anisotropic Stress-P_1_ and components of anisotropic stress, by region.

FE Result	Mid FC			Shoulder			IT		
	Slope	R^2^	*p* value	Slope	R^2^	*p* value	Slope	R^2^	*p* value
Anisotropic Stress-P_1_	2.364	0.703	<0.001	0.509	0.316	0.001	1.174	0.712	<0.001
Axial stress	2.353	0.695	<0.001	0.492	0.301	0.001	1.168	0.709	<0.001
Transverse stress	0.124	0.670	<0.001	0.059	0.509	<0.001	–	–	–
Shear stress	0.196	0.432	0.006	0.078	0.462	<0.001	–	–	–

Other anisotropic stresses, especially those in the shoulder region, had only weak or moderate correlations with isotropic Stress-P_1_ (low R^2^), suggesting fiber architecture caused the considerable changes to the anisotropic FE results. As fiber misalignment and dispersion were not strongly linked to FE results as individual parameters, the role of fiber structure in mechanics appears to be complex.

### Multivariate Regressions for Anisotropic Stresses

The multivariate regressions identified potential improvements to linear regression models of anisotropic stresses against isotropic Stress-P_1_ alone. In the mid FC, a multivariate regression using isotropic Stress-P_1_ and lipid area improved the regressions against transverse (R^2^ increased from 0.670 to 0.828) and shear stresses (R^2^ increased from 0.432 to 0.700). For the shoulders, a multivariate regression including FC thickness in addition to isotropic Stress-P_1_ improved regressions for anisotropic Stress-P_1_ (R^2^ increased from 0.316 to 0.424) and axial stress (R^2^ increased from 0.301 to 0.410). No other multivariate regressions for anisotropic stresses increased R^2^ by at least 0.1 compared to a linear regression against isotropic Stress-P_1_ only.

## Discussion

This study sought to quantify fiber orientation and dispersion in coronary atherosclerotic plaques and the impact of these fiber structures on the critical mechanical conditions when pressurized. We showed that fibers were more organized and more aligned with the local lumen boundary in the mid FC and IT, and less organized and less aligned in the plaque shoulder region (Table 2). In addition to the effect of local luminal curvature, such differences in fiber structure may contribute to the high stresses in the shoulder region where most FC ruptures (~60%) occur.^29^ In this study, high stress appearing in an area at least 2500 *µ*m^2^ was extracted for analysis. This threshold acted as a filter to avoid reporting very high elemental stress, which might be due to mesh distortion or local imperfect geometric configuration, e.g. sharp curvature. The criteria ignored about 15–30 of the highest-stress elements in each plaque region being equivalent to around the 95–99 percentile of stress in a plaque region. We also have calculated the 95 percentile stress and obtained the same conclusions.

Fiber groups often bifurcate around the lipid core in the shoulder region (Fig. 1a and 1b). Tangency is usually maintained with the lumen boundary and the lipid core, but the fibers are clearly not concentric in the 2D-plane as they are in healthy arteries. Seam-like features traverse from the lumen near the shoulders towards the ends of the lipid core, defining the border between fiber groups diverging to either side of the lipid core. These diverging fibers are highly misaligned and dispersed, as in the left shoulder of Fig. 1b. This may explain why fibers in the shoulder region had higher misalignment and dispersion. The seams follow a similar trajectory as the slender bands of high stress between the shoulder and lipid core, noted in the anisotropic model (Fig. 4b and 4c). The bands have highest stress at the lumen border of the shoulder, but extend deeper into the tissue and approach the border of lipid and fibrous tissue. Loads are passed along rather than adjacent to fibers. Considering the relatively weaker material^23^ at the border region induced by inflammation,^30^ this transmitted loading may damage local tissue^22^ and eventually lead to FC rupture from the interior to the surface.^9^,^12^ Alternatively, the bands may suggest a mechanism allowing fractures initiated at the lumen surface, where stresses are highest, to propagate along the bands to the interior.^13^

Elastin and collagen are the principal fibers represented in the plaque structure. Orientation of a fiber defines the direction in which the fiber is stiffest and strongest. Indeed, mechanical loading is mostly distributed along the fibers, with axial stress by far the highest anisotropic component and nearly as high as anisotropic Stress-P_1_ (Figs. 4 and 6). Most studies report a modulus of elastin in the range 0.3–1.2 MPa with a failure strain of up to 200%.^1^ Collagen has modulus of 600 ± 200 MPa, a failure strain of 13 ± 2%, and a failure stress of 60 ± 10 MPa.^51^ Considering the material strength and extensibility of the fibers, fracture of either elastin or collagen fibers is not expected under physiological conditions. The material structure between fiber bundles is weak relative to the fibers themselves^28^ and therefore has lower material strength.^35^ Anisotropic analysis revealed that transverse stress (stress pulling fibers apart or compressing fibers; Fig. 4d) was usually small (Fig. 6) and negative and likely contributes less to FC rupture. However, the pathological impact of compression in atherosclerosis remains unclear. Compared with transverse stress, shear stress was higher in the FC (Figs. 4e and 6), which may cause tissue sliding and tearing leading to structure failure. Experiments and clinical reports of failure modes plaque rupture are sparse and lack common terminology, but at least some suggest a role of delamination between fiber layers.^13^ This suggests fracture is the result of failure of the interfaces amongst fibers or between fibers and their surrounding matrix. Further, fiber damage commonly occurs in atherosclerotic plaques, including calcification of elastic fibers^2^ or cleavage of intact collagen fibers by matrix metalloproteinases.^24^ Fiber material strength and extensibility may decrease from these damages, permitting plaque rupture under physiological conditions due to the high stress along the fiber. More studies, in particular experimental studies, are needed to explore the relationship between the fiber alignment, pathological fiber features, and structure failure.

Mechanical analysis of human coronary plaques has been used to explain the location of plaque rupture.^11^,^25^,^47^ Such analysis shows potential as a novel tool to analyze the behavior of coronary plaques and can improve the ability of plaque imaging to predict different clinical presentations.^5^,^41^ A reliable modeling strategy is therefore needed for accurate and reproducible stress calculations. Including fiber structure caused a statistically significant change in Stress-P_1_ across the mid FC and IT regions, and individual plaques had considerable changes in predicted Stress-P_1_ when fiber orientation was included (Fig. 5). These data imply a need to depict the fiber structure *in vivo* for plaque-specific analysis, which would represent a significant challenge for current imaging techniques. An* ex vivo* preliminary study has shown the potential to characterize fiber orientation in the aorta with a 7 Tesla magnetic resonance system.^14^ Further development of emerging imaging techniques, such as polarization-sensitive optical coherence tomography,^27^ may enable the* in vivo* identification of fiber thickness and orientation in the future.

It has to be pointed out that high stress concentration does not necessary lead to plaque rupture and symptom. However, it has been demonstrated that high stress is associated with clinical symptoms^31^,^41^,^53^ and subsequent major adverse event^5^,^32^ in both coronary and carotid circulations. By comparing stress level obtained from computational analysis between ruptured and unruptured lesions, Cheng *et al*., suggested a rupture threshold of 300 kPa.^11^ In this study, 8 out of 16 isotropic model cases had stresses over 300 kPa in the key regions (all 8 in shoulder regions). With the anisotropic model, 7 of 16 cases had a region exceeding 300 kPa (6 shoulders and 2 mid FC, where both a shoulder and the mid FC in one case exceeded 300 kPa). Histology analysis with ruptured lesions showed that 37% (25/67) ruptures occurred in the mid FC and 63% in the shoulder region.^29^ In this study, most high stress concentrations were found to be located in the shoulder region in both isotropic and anisotropic models. This might be because histological slices used for analysis in this study had thick FC (2 cases with mean FC thickness between 100 and 200 *µ*m and the rest had FC thicker than 200 *µ*m). The location of stress concentration could switch from shoulder region to the mid FC when FC is thin (e.g., <200 *µ*m).^52^ Moreover, apart from stress level, the material strength of the local tissue is a dominating factor for rupture. It is mainly determined by pathological conditions in the tissue (e.g., inflammatory burden) and geometry (e.g., thickness). Ideally, the combination of stress level and local material strength should be used to evaluate the rupture risk. Using current technologies, the material strength cannot be obtained noninvasively, but may be assessed indirectly from tissue thickness and pathological features, characterised by* in vivo* imaging.^40^

Histological stain, including picrosirius red, H&E, and Masson’s trichrome, and other microscopy techniques, e.g., polarized microscopy, electron microscopy, and fluorescence microscopy multiphoton microscopy, can be used to visualize collagen fibers.^16^,^33^ In this retrospective study, histological sections were stained using H&E and EVG. Image processing was performed on slices stained by EVG harnessing its good contrast between elastin (in black) and collagen (in red) (Figs. 1 and 2); the one stained with H&E acted as a reference.

There are several limitations to this study. Firstly, fibers in different plaque regions and between different patients may have different material properties, which were not considered in the current analysis. Secondly, finite element models were constructed based on autopsy slides which could be subject to tissue damage and geometrical distortion during histological processing, despite our best efforts to minimize these. Thirdly, the number of subjects was small (sixteen plaques were analyzed), owing to the difficulty in collecting donors. Fourthly, static loading was used for the analysis. Micro-damage induced by the variation of loading during a cardiac cycle might accumulate resulting in material fatigue.^21^ This however was not considered in this study. Fifthly, the residual stress was not considered. Finally, analysis was limited to a 2D plane and the effect from blood flow was not considered.

In conclusion, significant differences were found in fiber architecture along the luminal region of human coronary atherosclerotic plaques. Fibers in the shoulder regions were more dispersed and less aligned with the lumen than those in the mid FC and IT regions. Considerable levels of shear stress exist in the FC, which may cause delamination between fibers, ultimately leading to FC rupture.

## Electronic supplementary material

Below is the link to the electronic supplementary material.

Supplementary material 1 (DOCX 1014 kb)
