# Supplementary material for: Impact of Fiber Structure on the Material Stability and Rupture Mechanisms of Coronary Atherosclerotic Plaques
Source: Ann Biomed Eng. 2017 Mar 30;45(6):1462–74. doi: 10.1007/s10439-017-1827-3 (PMC5415591; doi:10.1007/s10439-017-1827-3)
Supplement: Supplementary file 1 — Supplementary material 1 (DOCX 1014 kb) [file 10439_2017_1827_MOESM1_ESM.docx]

**Supplemental Material**

Supplement to: Douglas GR et al., Impact of fiber structure on the material stability and rupture mechanisms of coronary atherosclerotic plaques

**Technical Details of Methodology (from 2.2 Image Processing)**

*Canny Edge Detection of Fibers*

Canny edge detection uses a 1-D Gaussian filter, *H*, to smooth the original histology image,

$$H=\frac{1}{\sqrt{2\pi}\sigma^{2}}exp\left( -\frac{k^{2}}{2\sigma^{2}} \right)$$

in which *σ* is the standard deviation (set to be in this study), and *k* is the span of the filter (set to be [-5, 5] pixels; each pixel is about 3.5µm). The filter was applied vertically and horizontally to remove noise and speckle in the image. To detect edges at various angles, the gradient of the filtered image were calculated vertically and horizontally. Two thresholds were used to set values of the gradient magnitude to define edges: high (strong edges) and low (weak edges). The high threshold was selected as the gradient value such that 30% of pixels in the image were edges. The low threshold was set at 40% of the gradient value set as the high threshold. Pixels above the low threshold *and* adjacent to a high threshold edge were also considered edges. The edge detection method was verified by overlaying these detected fibers on the original image of the plaque and testing on images of synthetically generated fibers.

*Orientation Assignment for Fiber Angles*

Connected adjacent pixels of edges (assumed to be fibers) were found using the *regionprops* algorithm in Matlab (The MathWorks, Inc., MA, USA), considering adjacent and diagonal pixels (8-connectivity). Fibers containing four or fewer connected pixels were removed, as they are too small to give reliable fiber properties. Fibers larger than 500 connected pixels were partitioned into smaller features by overlaying a 75×75 grid, which removes the pixels from the fibers intersecting the grid.

The local orientation of each connected fiber was calculated by the *regionprops, orientation* algorithm in Matlab^18^. This method calculates the second moments of area for each fiber (*I*, *I*, and *I*) and *θ_fiber_*, the Cartesian angle of the a fiber’s major axis relative to the horizontal. If *I* is less than *I*, the orientation angle can be calculated using,

$$\theta_{fiber}=atan\left( \frac{\left| I_{xx}-I_{yy} \right|+\sqrt{\left| I_{xx}-I_{yy} \right|^{2}+4I_{xy}^{2}}}{2I_{xy}} \right)$$

in which $\theta_{fiber}\in\left[ -{90}^{^{\circ}}, {90}^{^{\circ}} \right]$.

*Mapping Fiber Angles to the Surrounding Geometry*

Fiber orientation for each pixel in the sample, *θ_µ_*(*x,y*), was mapped using the circular mean of orientation of the fibers within a 51×51 pixel region of interest (ROI),

$$\theta_{\mu}=\frac{1}{2}atan2\left( \frac{\sum_{i,j=-25}^{25} sin\left( 2\theta_{fiber}\left( i,j \right) \right)}{n},\frac{\sum_{i,j=-25}^{25} cos\left( 2\theta_{fiber}\left( i,j \right) \right)}{n} \right)$$

in which $\theta_{\mu}\in\left[ -{90}^{^{\circ}}, {90}^{^{\circ}} \right]$ and *n* is the number of pixels in the ROI having an assigned orientation. A circular mean, rather than a standard mean, is required because fiber orientation is bidirectional (e.g., 0° ≡ 180° and +90° ≡ -90°). The method was validated against images with simulated fibers at known angles. This mapping assigns orientation to spaces between the fibers (required for the continuum finite element models) and removes highly misaligned fibers, which are typically minor artifacts from histological preparation.

*Referencing Orientation to the Artery Coordinate System*

To give context to the fiber orientation, an assumed orientation is needed as a reference. In healthy arteries, fibers are predominantly aligned in the circumferential direction and the lumen is approximately circular. Moreover, for a healthy artery, the geometry can be assumed to be symmetrical. A circular coordinate system can therefore be used, where fibers are expected to traverse circumferentially but not radially.

Arteries with atherosclerotic disease are generally asymmetrical. A tangential reference instead of a circular one was used in this study to deal with the irregular geometry. Local orientations along the lumen and outer wall boundary of the plaque, *θ_ref,contour_*(*x_i_*,*y_i_*), were calculated as tangents to points defining the contour *p_x_*_,_*_y_*,

$$\theta_{ref contour}\left( x_{i},y_{i} \right)=atan\left( \frac{p\left( y_{i+1} \right)-p\left( y_{i} \right)}{p\left( x_{i+1} \right)-p\left( x_{i} \right)} \right)$$

in which $\theta_{ref contour}\in\left[ -{90}^{^{\circ}}, {90}^{^{\circ}} \right]$. The fiber orientation of the samples used in this study was generally tangential, especially in the region with intimal thickening (IT). The tangential orientations of the lumen and outer wall were linearly interpolated through the cross-section using the *griddata* function in Matlab to define a reference orientation, *θ_ref_*(*x_i_*,*y_i_*). The unsigned difference between the Cartesian imaged orientation (*θ_µ_*) and assumed reference orientation (*θ_ref_*) gives the misalignment of fiber orientation (*θ_i_*) as

$$\theta_{i}=\left| \theta_{\mu}-\theta_{ref} \right|$$

in which $\theta_{i}\in\left[ 0^{^{\circ}}, {90}^{^{\circ}} \right]$.

*Fiber Dispersion*

Degree of fiber dispersion, *κ*, may affect the material properties of the tissue. Fiber dispersion was described previously in a constitutive material model for fibrous tissues to quantify scatter of fibers^16^

$$\kappa=\frac{1}{4}\int_{0}^{\pi} \rho\left( \theta\right){sin}^{3}\theta d\theta$$

in which *θ* is a bin of fiber orientations, relative to their mean orientation, *θ_µ_*, at the center of the ROI, and *ρ(θ)* is the normalized density distribution of fiber orientation in each bin. In this study, fiber orientations were assigned into twenty evenly-spaced bins and *κ* was calculated in a 51×51 pixel ROI. Perfectly parallel fibers corresponds to *κ*=0 and an even scatter of fibers (a continuous material) has *κ*=1/3 (see **Figure 2** in the manuscript for representative fibers showing a range of dispersion values).

| 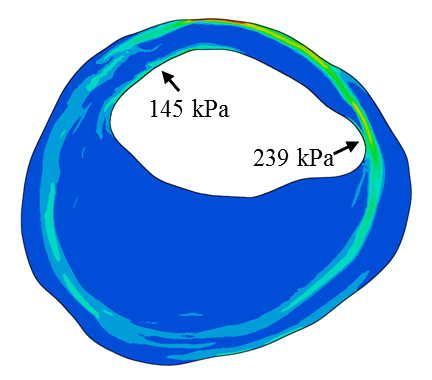 | 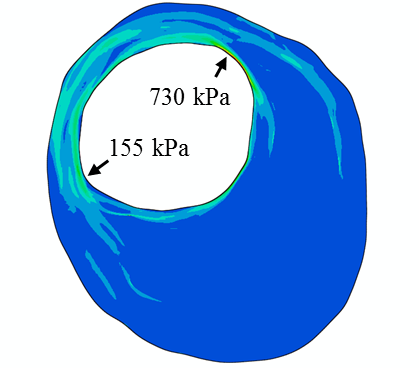 |
| --- | --- |
| 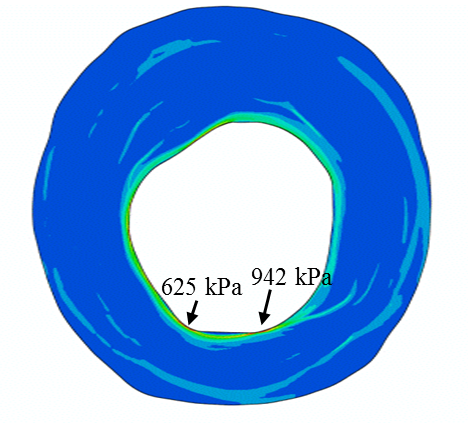 | 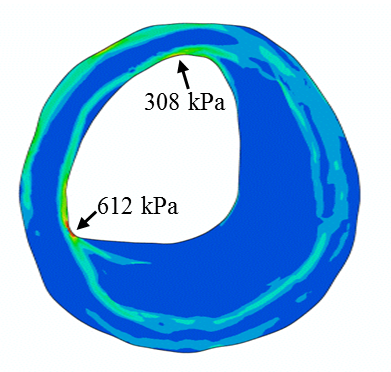 |
| 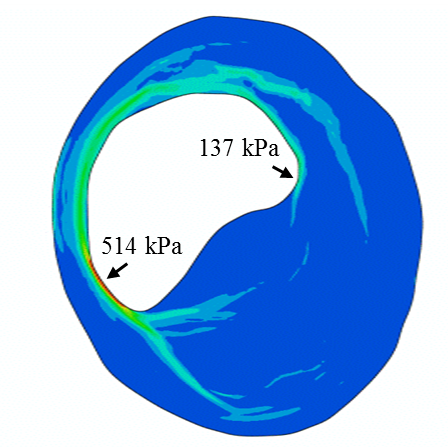 | 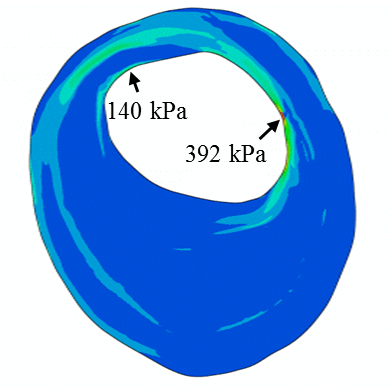 |


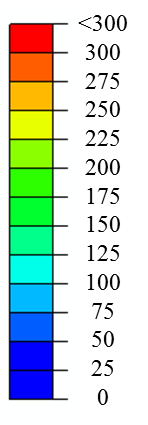


Figure S1. Representative models showing slender bands of Stress-P_1_ extended deeper into the structure; unit: kPa.
